# Factors associated with receipt of a timely infant birth dose of hepatitis B vaccine at a tertiary hospital in North-Central Nigeria

**DOI:** 10.1371/journal.pgph.0001052

**Published:** 2022-09-12

**Authors:** Florence O. Bada, Kristen A. Stafford, Sophia Osawe, Eleanor Wilson, Nadia A. Sam-Agudu, Hegang Chen, Alash’le Abimiku, James D. Campbell

**Affiliations:** 1 Department of Epidemiology and Public Health, Graduate Program in Life Sciences, University of Maryland School of Medicine, Baltimore, MD, United States of America; 2 International Research Center of Excellence, Institute of Human Virology Nigeria, Abuja, Nigeria; 3 Division of Epidemiology and Prevention, Institute of Human Virology, University of Maryland School of Medicine, Baltimore, MD, United States of America; 4 Department of Epidemiology and Public Health, University of Maryland School of Medicine, Baltimore, MD, United States of America; 5 Plateau State Human Virology Research Center, Plateau State, Nigeria; 6 Division of Clinical Care and Research, Institute of Human Virology, University of Maryland School of Medicine, Baltimore, MD, United States of America; 7 Department of Pediatrics, Center for Vaccine Development and Global Health, University of Maryland School of Medicine, Baltimore, MD, United States of America; University of Michigan, UNITED STATES

## Abstract

The World Health Organization recommends universal vaccination of medically stable infants with Hepatitis B vaccine within 24 hours of birth to prevent mother-to-child transmission of Hepatitis B virus (HBV) infection. However, the proportion of infants who receive a timely birth dose is extremely low in Nigeria. We reviewed the implementation of an infant HBV vaccine schedule at a single center and identified factors affecting the receipt of a timely birth dose of HBV vaccine. We conducted a retrospective cohort study utilizing data from the INFANT study, a 2013–2017 prospective cohort study of pregnant women with and without HIV and their infants We utilized bivariate and multivariable logistic regression to assess if maternal characteristics, or the day of the week on which the infant was born were significantly associated with timely receipt of a birth dose of HBV vaccine. Receipt of HBV vaccine on the day of birth or the following calendar day were considered a timely birth dose. Among 409 infants in our cohort, 133 infants (33%) received a timely birth dose of HBV vaccine. Only the day of the week on which infants were born was significant (p<0.0001): when compared to Friday, infants born Monday through Thursday had significantly higher odds of receiving a timely birth dose, while infants born on a Saturday or Sunday had similar (low) odds. We found no association between maternal age, education, marital status, HIV status, parity and mode of delivery, and infant receipt of a timely birth dose of HBV vaccine. National immunization programs could improve timely HBV birth dose rates by providing access to vaccine immediately following birth at all infant delivery venues on all days of the week. Where not possible, there should be rapid linkage to the nearest facility where HBV vaccination is immediately available.

## Introduction

Globally, approximately 257 million people are chronically infected with hepatitis B virus (HBV) [1]. Chronic HBV infection is associated with considerable morbidity and mortality [2]; 15–40% of people with chronic HBV infection go on to develop liver cirrhosis, liver failure, or hepatocellular carcinoma and 15–25% die from HBV-related liver disease [3]. In sub-Saharan Africa, the risk of MTCT of HBV without intervention among mothers with hepatitis B surface antigen (HBsAg), a biomarker of active infection, and hepatitis B e antigen (HBeAg), a biomarker of active infection with high infectivity, has been estimated at 38.3%, and for mothers with HBsAg alone, 4.8% [4]. This is reflected in 1% of newborns in sub-Saharan Africa; approximately 370,000 infants, infected with HBV via MTCT every year [4]. While HBV infection in adulthood leads to chronic HBV infection in less than 5% of adults, infants infected in the perinatal period have a 90% risk of developing chronic HBV infection [5, 6]. In West Africa, infants who are infected with HBV through MTCT have been found to have five times the risk of liver fibrosis as compared to people infected later in life via horizontal transmission [7]. Nigeria is Africa’s most populous country, with a population of 211 million people [8] and a high endemicity of HBV. The seroprevalence of HBsAg in Nigeria is approximately 8.1% [9], translating to over 17 million people living with chronic HBV infection. HBsAg seroprevalence in pregnant Nigerian women is intermediately high at approximately 6% [9, 10].

Several strategies and interventions have been recommended to prevent MTCT of HBV [11–13]. These include timely identification of infected mothers and exposed infants, with provision of a birth dose of HBV vaccine and hepatitis B-immune globulin to exposed infants within 12 hours of birth where feasible [11, 12] Furthermore, pregnant women with HBV viral load ≥ 200,000 IU/mL benefit from antiviral agents which decrease their viral load and reduce risk of MTCT to their infants [13]. However, the World Health Organization recommends universal vaccination of all medically stable infants regardless of their mother’s HBV status [14]. Each infant should initiate three- or four-doses of HBV vaccine as close to birth as possible to minimize MTCT of HBV and proffer life-long protection from HBV infection [14, 15].

Of the 46 countries in sub-Saharan Africa, Nigeria is one of only 11 offering a vaccine schedule that includes a birth dose of HBV vaccine [16]. However national coverage with a birth dose of HBV vaccine was only 52% in 2019 [17] and the proportion of infants who receive a timely birth dose of HBV vaccine, a vaccine received within 24 hours of birth, ranges between 13 and 27% [18–20]. We reviewed the implementation of an infant HBV vaccine schedule at a tertiary hospital in North-Central Nigeria and identified factors affecting the receipt of a timely birth dose of HBV vaccine.

## Materials and methods

We conducted a retrospective cohort study utilizing data from the INFANT (Innate, Adaptive and Mucosal Immune Responses in HIV-1 Exposed Uninfected Infants: A Human Model to Understand Correlates of Immune Protection) study. INFANT was a prospective cohort study of mother-infant pairs at a tertiary hospital in North-Central Nigeria enrolled between 2013 and 2017 [21].

### Ethics statement

Approval for the use of de-identified data from the INFANT study was provided by the University of Maryland Baltimore Institutional Review Board, and the Plateau State Specialist Hospital in Nigeria. For this study, consent was waived because it had been obtained previously during the parent study for the storage and use of stored samples in subsequent studies. Contacting patients to obtain additional consent would have been the only reason to utilize patient identifying information. Linking identifying information and contacting patients to obtain consent is associated with a risk of breach of confidentiality. This was avoided by obtaining a waiver of consent.

#### Inclusivity in global research

Additional information regarding the ethical, cultural, and scientific considerations specific to inclusivity in global research is included in (S1 Checklist).

### Study setting

Nigeria added HBV vaccine to its National Programme of immunization in 1995, but the vaccine was not widely available until 2004 [22]. At the time of the INFANT study, the approved HBV vaccine schedule consisted of three doses of monovalent HBV vaccine to be given to infants at birth, and at six and 14 weeks of life [18]. Infant vaccinations were given at an immunization clinic in the pediatric unit of Plateau State Specialist Hospital, which provided services on Tuesdays and Thursdays of every week.

### Parent study

Pregnant women living with and without HIV were offered enrollment into the INFANT study as they attended antenatal clinic. At the time of registration for antenatal care, all pregnant women were tested for HIV as part of standard of care [23]. For women recruited into the study who tested HIV-negative at the time of registration for antenatal care, the INFANT study procedures included repeat HIV testing between week 32 of gestation and delivery. Eight study follow-up visits occurred at 1, 4, 7, 10, 15, 24, 36 and 52 weeks after delivery.

#### Inclusion criteria

Participating pregnant women were to be at least 18 years of age, to have voluntarily chosen to breastfeed their infants and able to meet the study assessment schedule.

#### Exclusion criteria

Prospective participants were excluded if they had complications during pregnancy or delivery (e.g., eclampsia, chorioamnionitis), if the subsequently delivered infant had neonatal asphyxia, seizures, or sepsis; if infant gestational age was less than 36 weeks at birth, or if infant birth weight was less than 2.5 kg. All infants were tested for HIV via DNA polymerase chain reaction at birth per study protocol, and between 4–6 weeks of life per standard of care, and any infants found to be HIV-infected were also excluded from the study [21].

#### Study procedures

Questionnaires detailing maternal socio-demographic, clinical and obstetric factors, and infant birth, feeding, clinical and vaccination data were completed by research personnel via maternal participant interviews and medical record abstraction. Physical examinations of infants were performed by pediatricians and pediatric nurses on the research team, and blood, stool, saliva and breast milk collected by laboratory personnel on the research team at each study visit. The blood samples were processed and plasma aliquoted and stored in -80°C freezers within six hours of blood collection.

### Study participants

All participants enrolled in the INFANT study were eligible for inclusion in our study. A de-identified data set containing maternal demographic information, maternal obstetric history, infant anthropometric and vaccination data from the INFANT study was obtained in line with the ethical provisions for such transfers.

#### Exclusion criteria

We excluded all second twins (n = 10) from our analysis.

### Statistical analysis

We determined the proportion of infants who received each of the birth, six-, and 14-week HBV vaccines (HBV1, HBV2 and HBV3) and the timing of HBV1 vaccine. The primary outcome measure was the proportion of infants who received a timely birth dose of HBV1 vaccine. We described the birth dose as a dose received within the first seven calendar days of life and a timely birth dose, as a dose given on the day of birth or the following calendar day.

We conducted bivariate analysis using Pearson’s chi-square, Fisher’s, and t-tests as indicated to determine the association between covariates of interest and a timely birth-dose of HBV1 vaccine. We used this to identify variables to evaluate in multivariable logistic regression but adjusted for all variables identified a priori as potential confounders. Covariates included maternal factors thought to influence a mother’s decision to vaccinate her child or affect her ability to present her child for HBV vaccination at the clinic. These factors included maternal age, education, marital status, HIV status and parity. We also analyzed factors that may have incapacitated a mother and rendered her unable to present her child for vaccination soon after delivery, such as her mode of delivery; cesarean or vaginal. In view of the immunization clinic schedule of services (Tuesdays and Thursdays only), we evaluated the effect of the day of the week the infant was born on receipt of a timely birth dose. All analyses were conducted using SAS version 9.4.

## Results

A total of 409 pregnant women and 419 infants had been recruited into the INFANT study. For our analysis, we included 409 mother-infant pairs from the INFANT study after excluding one infant each from ten pairs of twins. Nearly all infants (404/409, 99%) received HBV1; 95% (390/409) received HBV1 and HBV2 and 94% (384/409) received HBV1, HBV2 and HBV3 vaccines. Among the 404 infants that received HBV1, 91% (366) received a birth dose (within seven days of birth), yet only 33% received a timely birth dose of HBV1 vaccine.

Maternal and infant factors associated with infants who received of a timely birth dose of HBV1 vaccine as compared to those that did not are summarized in Table 1. The two groups were similar in terms of maternal demographic characteristics such as maternal age, educational status, marital status and occupation, and maternal obstetric history including parity, mode of delivery and HIV status (Table 1). However, the two groups differed in terms of day of the week on which infants were born, (Table 2). When compared to Friday which is the day farthest away from the next vaccination clinic, infants born on a Monday had 152.6 (95% CI 31.57–737.61) times the adjusted odds of having a timely birth dose, those born on a Tuesday, Wednesday, or a Thursday had 16.7 (95% CI 3.69–75.78), 78.9 (95% CI 17.01–366.09) or 9.0 (95% CI 1.91–42.37) times the odds of having a timely birth dose. While infants born on a Saturday or Sunday were similar to infants born on a Friday with 0.4 (95% CI 0.04–4.69) and 1.3 (95% CI 0.17–9.59) times the adjusted odds of having a timely birth dose.

**Table 1:** Comparison of maternal and infant characteristics of infants who received a timely birth dose of HBV vaccine with those that did not.

	All	Timely Birth dose	Untimely Birth dose	
Characteristics	N = 404	n = 133 (row %)	n = 271 (row %)	P
*Maternal*				
Maternal Age (yr), Median (IQR)	30 (27–34)	30 (27–34)	31 (27–34)	0.96
HIV Status,				
Positive n (%)	299	99 (33.1)	200 (66.9)	0.89
Marital Status				
Married, n (%)	390	128 (32.8)	262 (67.2)	0.78*
Education				0.63
Elementary or less	134	42 (31.3)	92 (68.7)	
At least secondary	270	91 (33.7.0)	179 (66.3)	
Occupation				
Employed, n (%)	272	83 (30.5)	189 (69.5)	0.14
Primiparous, n (%)	56	17 (30.4)	39(69.6)	0.66
Mode of Delivery				
Vaginal, n (%)	366	123 (33.6)	243 (66.4)	0.36
Previous Pregnancies, n (%)				0.91
0	56	17 (30.4)	39 (69.6)	
1–3	252	84 (33.3)	168 (66.7)	
≥ 4	96	32 (33.3)	64 (66.7)	
*Infant*				
Day of birth, n (%)	(N = 403)	(n = 133)	(n = 270)	< .0001
Monday	62	52 (83.9)	10 (16.1)	
Tuesday	60	22 (36.7)	38 (63.3)	
Wednesday	57	41 (71.9)	16 (28.1)	
Thursday	54	13 (24.1)	41 (75.9)	
Friday	61	2 (3.3)	59 (96.7)	
Saturday	64	1 (1.6)	63 (98.4)	
Sunday	45	2 (4.4)	43 (95.6)	

* Fisher’s Exact Test

**Table 2:** Multivariable analysis of the association between day of birth and the odds of receiving a timely birth dose of HBV vaccine.

	Unadjusted OR (95% CI)	Adjusted OR* (95% CI)
Day of Birth		
Friday	Reference	Reference
Monday	153.400 (32.13–732.41)	152.60 (31.57–737.61)
Tuesday	17.08 (3.80–76.84)	16.73 (3.69–75.78)
Wednesday	75.59 (16.48–346.68)	78.90 (17.01–366.09)
Thursday	9.35 (2.00–43.68)	8.99 (1.91–42.37)
Saturday	0.47 (0.04–5.30)	0.41 (0.04–4.69)
Sunday	1.37 (0.19–10.13)	1.29 (0.17–9.59)

*Adjusted for maternal HIV status, marital status, educational status, parity, and mode of delivery

## Discussion

In our study cohort, the proportion of infants who received the full 3-dose regimen of HBV vaccine was very high; much higher than the Nigerian national coverage for infant HBV in 2019, which were, 52% for birth dose and 57% for all three doses [17]. This was likely due in part to the frequency and timing of study visits for the INFANT study, which were designed to coincide with the timing of required infant vaccinations and included maternal health education on infant and self-care. These high rates of coverage are likely to prevent HBV infection later in life. However, only 33% of infants in our study cohort received a timely birth dose of HBV1 vaccine; a birth dose before their third calendar day of life which is key to preventing MTCT of HBV infection. This pattern of a high proportion of infants receiving HBV1 vaccine, with only a fraction falling within the desired timeframe has also been reported in other studies in Nigeria. A cross sectional study which included 23 health facilities in Nigeria reported low coverage with HBV1 birth dose of 23% (range: 12–40%) and timely birth dose coverage of 13% [19]. In a study in South East Nigeria, 93.8% of infants received HBV1 but only 19.2% within the first 2 weeks of birth [18]. In yet another study in the same region of Nigeria, the mean age at receipt of HBV1 was 28 days (SD 20.4) [24]. These results are similar to those from The Gambia; though 84–93% of infants in The Gambia received a birth dose of HBV1, only 1–9% had it in a timely fashion [19, 25]. However, in Botswana, 94% (range: 80–100%) of infants received a birth dose and 74% (range: 57–88%) received a timely birth dose [19].

Several factors could influence the timely receipt of an infant’s birth dose of HBV1 vaccine. Maternal factors such as marital status may reflect availability of (partner) support following delivery. Another important factor is if the index pregnancy was the mother’s first pregnancy. In such cases, the new mother may be unaware of all the services required for her newborn and may miss some services or obtain them late. Mothers who delivered by cesarean section are more likely to be incapacitated post-delivery, and thus may not obtain newborn services in a timely manner. However, in our study, none of the maternal factors we considered were significantly associated with the timely receipt of an infant’s birth dose of HBV1. Other studies identified maternal educational status to be associated with the odds of receipt of HBV1 vaccination within the first week of life [25, 26]. These studies however, included infants born at home and the educational status of mothers who deliver at home is likely to differ substantially from those in our study who delivered in a health facility and agreed to participate in a research study.

On evaluating infant factors, the day of the week the infant was born was significantly associated with the odds of receiving a birth dose of HBV1 vaccine. Infants born on Friday, Saturday and Sunday had similar (low) odds of receiving a timely birth dose of HBV1 vaccine; however, infants born on Monday, Tuesday, Wednesday, and Thursday had much higher odds of receiving a timely birth dose of HBV1 vaccine when compared to infants born on Fridays. This is not surprising, considering HBV vaccine was administered to infants only on Tuesdays and Thursdays at the study facility. This reinforces findings from previous studies in other African countries that sought to identify barriers to the receipt of a timely birth dose of HBV1 vaccine. These studies identified that the lack of daily vaccination services played a significant role in timely receipt of a birth dose of HBV1 vaccine [19, 26]. Facilities in similar LMIC countries like Nigeria that reported high coverage with a birth dose of HBV1 offered daily vaccination services including weekends and public holidays and vaccinated babies in delivery wards at the time of birth as part of essential newborn services [19].

We were unable to utilize the standard definition for a timely birth dose of HBV vaccine (a dose given within 24 hours of birth) [14] because our study utilized data from the INFANT study which only recorded the day of vaccination, not the time. Also, due to exclusion criteria for INFANT, several infant factors that could potentially influence the receipt of a timely birth dose of HBV1 vaccine could not be evaluated. These include infants with birth weight less than 2.5 kg, and infants with neonatal asphyxia, seizures, or sepsis. We were, however, able to evaluate the effects of several maternal factors and a gap we identified in health service delivery.

## Conclusion

Even though 91% of infants received a birth dose of HBV vaccine, only 33% received a timely birth dose, which is key to preventing MTCT of HBV. The availability of daily child vaccination services seems to be an obvious requirement for improving access to the birth dose of HBV vaccine. However, this study provides evidence of how, and to what extent this affects vaccine uptake. In the absence of daily services, infants in our cohort faced vastly different odds of HBV vaccine uptake simply based on the day of the week on which they were born. National immunization programs could likely improve timely HBV1 birth dose rates by providing access to vaccine immediately following birth at all infant delivery venues on all days of the week. Where not possible, there should be rapid linkage to the nearest facility where HBV vaccination is immediately available.

## Supporting information

S1 Checklist Inclusivity in global research questionnaire. (DOCX)

S1 Data Timely hepatitis B birth dose study. (XLSX)
